# Morphological Autoencoders for Beat-by-Beat Atrial Fibrillation Detection Using Single-Lead ECG

**DOI:** 10.3390/s23052854

**Published:** 2023-03-06

**Authors:** Rafael Silva, Ana Fred, Hugo Plácido da Silva

**Affiliations:** 1 Department of Bioengineering (DBE), Instituto Superior Técnico (IST), Av. Rovisco Pais 1, 1049-001 Lisboa, Portugal; 2 Instituto de Telecomunicações (IT), Av. Rovisco Pais 1, Torre Norte—Piso 10, 1049-001 Lisboa, Portugal

**Keywords:** atrial fibrillation detection, ECG morphological features, supervised autoencoder, automatic feature extraction

## Abstract

Engineered feature extraction can compromise the ability of Atrial Fibrillation (AFib) detection algorithms to deliver near real-time results. Autoencoders (AEs) can be used as an automatic feature extraction tool, tailoring the resulting features to a specific classification task. By coupling an encoder to a classifier, it is possible to reduce the dimension of the Electrocardiogram (ECG) heartbeat waveforms and classify them. In this work we show that morphological features extracted using a Sparse AE are sufficient to distinguish AFib from Normal Sinus Rhythm (NSR) beats. In addition to the morphological features, rhythm information was included in the model using a proposed short-term feature called Local Change of Successive Differences (LCSD). Using single-lead ECG recordings from two referenced public databases, and with features from the AE, the model was able to achieve an F1-score of 88.8%. These results show that morphological features appear to be a distinct and sufficient factor for detecting AFib in ECG recordings, especially when designed for patient-specific applications. This is an advantage over state-of-the-art algorithms that need longer acquisition times to extract engineered rhythm features, which also requires careful preprocessing steps. To the best of our knowledge, this is the first work that presents a near real-time morphological approach for AFib detection under naturalistic ECG acquisition with a mobile device.

## 1. Introduction

Atrial Fibrillation (AFib) is a Cardiovascular Disease (CVD) characterized by the uncoordinated activity of the heart chambers, with the atria exhibiting irregular and high-rate electrical activity. It is the most frequent type of cardiac arrhythmia in the Western World, with an estimated prevalence of 46.3 million individuals worldwide [1,2], and it is one of the leading causes of stroke, as it increases the risk of having one by four to five times [3]. In addition, recent reports suggest that both global incidence and prevalence are on the rise, putting a greater strain on healthcare systems [4,5].

AFib occurs because of a lack of synchrony in atrial contraction since disorganized electrical impulses propagate throughout both atria. This can be caused by the appearance of ectopic pacemaker foci, or by structural and biochemical changes that delay regular electrical pulses [6]. Over time, risk factors and lack of treatment can deteriorate the atria’s condition, and AFib can progress from a paroxysmal to a permanent state, in a process known as atrial remodeling [7,8]. Because persistent AFib is linked to a higher risk of a severe stroke [9], AFib early diagnosis and monitoring are crucial to prevent further complications.

**Figure 1:**
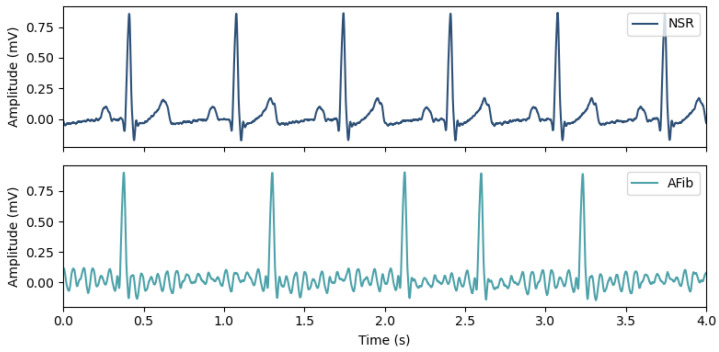
Morphological and rhythm changes between ECG recordings in Normal Sinus Rhythm (**top**) and in Atrial Fibrillation (**bottom**), where the absence of P-waves and the irregular heart rate are noticeable.

Diagnosing AFib is commonly made using the Electrocardiogram (ECG) to monitor the heart’s electrical activity (Figure 1). Both its rhythm and waveform morphology are changed by the presence of an irregular heart rate, the replacement of P-waves by fibrillatory waves (F-waves), and the lack of an isoelectric baseline [10].

Since one cannot predict rare AFib events to occur while at the hospital, continuous monitoring of patients at risk can be achieved in other environments (e.g., home and/or ambulatory) by using implantable cardiac monitors, wearable patch monitors, and Holter monitoring systems [11,12,13]. Cardiovascular monitoring has also been extended to the general population using wrist-worn wearable devices, which can benefit from automatic detection of heart conditions [14]. More recently, off-the-person devices, increasingly dubbed as “invisibles”, are integrated with everyday use objects and do not require a conscious effort to perform data acquisition, providing a more pervasive way to detect CVDs [15,16,17,18].

Because of the overwhelming amount of data generated by these long-term acquisitions, many Computer-Aided Diagnosis (CAD) tools have been developed over the last decades to assist in AFib detection. State-of-the-art CAD examples include algorithms based on Artificial Neural Networks (ANNs), Support Vector Machines (SVMs), and Decision-Trees (DTs) [19]. Most of these algorithms rely on ventricular and signal features extracted from ECG recordings, such as RR-Intervals (RRIs) and associated variability metrics, frequency content, among others. However, despite AFib being an atrial phenomenon, very few algorithms rely on atrial features (e.g., atrial wave morphology), mainly because of the relatively low amplitudes of the atrial activity and high sensitivity to noise [19].

Given the large number of features that can be extracted, feature selection can be computationally expensive (given that it is an NP-hard problem), and, depending on the number of chosen features, feature extraction can compromise the algorithms’ responsiveness. This way, algorithms that do not rely on feature engineering (e.g., only using morphology) or that use optimized features for a specific problem (e.g., using automatic feature extraction tools) can be advantageous for real-time (or near real-time) and resource-limited systems.

In addition, the duration of the temporal window in which the features are to be extracted has to be considered. Ideally, systems for real-time implementation need to have low latency between acquisition and result. Consequently, short-term features are preferred in monitoring systems. In this regard, morphological characteristics could be more efficient than ventricular and signal features, which frequently require more samples to become relevant [20].

To deal with such challenges, we propose an approach based on Autoencoders (AEs) to distinguish AFib from Normal Sinus Rhythm (NSR) in a beat-by-beat fashion. The AE is capable of automatically generating features from the morphology of the ECG waveforms, which can then be used by a standard Machine Learning (ML) classifier. To this end, two PhysioNet [21] databases were used to evaluate its performance: the MIT-BIH Atrial Fibrillation (AFDB) [22] and the Computing in Cardiology Challenge 2017 (CinC2017) [23]. In addition, we also examine the impact of Supervised AEs (SupAEs) and the effect of incorporating a short-term engineered ventricular feature on the overall classification performance.

To do so, this paper first briefly describes comparable state-of-the-art approaches for AFib detection (Section 2). Next, the methodology used to build the AE-based model is presented, along with the training approach (Section 3). Finally, the results and key findings are described (Section 4), compared to other approaches, and discussed (Section 5 and Section 6).

## 2. Related Work

**Figure 2:**
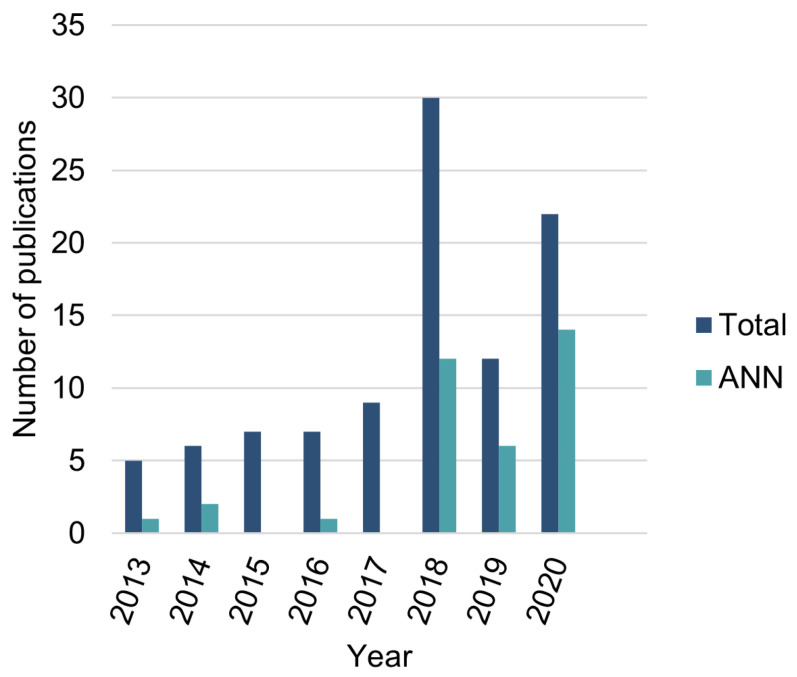
Number of AFib detection algorithm publications from 2013 to 2020, showing how the number of ANN-based algorithms have gained popularity over time. The year 2018 was boosted by the CinC2017 challenge. Data obtained from the Appendix A, Supplementary Data section of [19].

As stated in Section 1, many approaches around AFib detection have been developed over the years [19], where the use of ANNs has recently been more pronounced and achieves the best performances (Figure 2). There is a wide variety of algorithms based on ANNs, such as using Convolutional Neural Networks (CNNs), Deep Neural Networks (DNNs), Recurrent Neural Networks (RNNs), and AEs. The following subsections provide some insight into the current state-of-the-art of these approaches, taking into account the nature of the proposed model.

### 2.1. Without Feature Engineering

Feature extraction and feature selection are two of the main ML tasks since their purpose is to provide algorithms with meaningful and compact data. In AFib detection, Heart-Rate Variability (HRV) metrics are often used to measure the irregularity of RR-Intervals [19,24]. Examples of such ventricular features include: Root Mean Square of Successive Differences (RMSSD), Poincaré plots, Sample Entropy (SampEn), Turning Point Ratio (TPR), and Lyapunov exponents. In addition, signal features can include signal power, frequency content, statistical measures, Wavelet Transform, phase-space analysis, among others.

Using a Deep Learning (DL) approach, Baalman et al. [25] developed a feedforward neural network focused on classifying ECG beats (i.e., single cycle) as NSR or AFib. This approach demonstrated the capacity of DL models to automatically extract adapted features to a specific classification task. In addition, the authors used an attention mechanism to highlight the model’s hidden features, thus providing explainable results (in contrast with the typical black-box paradigm). Using their own ECG data recorded at 500 Hz, the authors achieved an accuracy of 96% and an F1-score of 94% using Lead II ECG recordings and, using Lead I recordings, the model achieved an accuracy of 93% and an F1-score of 90%.

Based on computer vision strategies, Fan et al. [26] proposed a fusion of two deep convolutional neural networks (DCNNs) with different filter sizes. Using ECG signals in one dimension, the output of both DCNNs is concatenated and fed into a set of 3 fully connected layers that produce an output for the AFib/NSR classification with a Softmax activation function. This model was tested in single-lead short ECG recordings from the CinC2017 dataset, and it achieved a 96.99% accuracy using a 5 s time window, and a peak accuracy of 98.13% using a 20 s input sequence.

To tackle the limitation of CNN-based models to analyze variable-duration ECG recordings, Zhang et. al [27] proposed a time-adaptive structure that consists of multiple densely connected CNNs (DenseNets) and a Bidirectional Long Short-Term Memory (Bi-LSTM) cell. Using 10 s cropped single-lead ECG fragments, the binary classification performance was 99% and 87% for the CinC2017 and AFDB datasets, respectively.

### 2.2. Autoencoder-Based

AEs are well-known for their ability to perform dimensionality reduction and have been used in numerous applications. However, their usage for AFib detection has been very scarce.

Yuan et al. [28] developed an approach for AFib detection from ECG records using a stacked Sparse Autoencoder (SAE) based on 84 selected features extracted from the RR-Intervals and P-wave measurements within a 10 s ECG window. The AE used to achieve data compression had 84 input nodes and 2 hidden layers with 300 nodes each. AFib detection was made by stacking a Softmax activation function to the extracted features of the AE. Using ECG recordings from the MIT-BIH databases, the model first achieved a detection accuracy of 75.6%, and, after fine-tuning the model, a 98.3% accuracy was reported.

A similar approach was followed by Chen and Ying [29], where a stacked SAE receives 19 features extracted from the ECG records, including statistical measures, parameters from the Hilbert–Huang transform, and Wavelet decomposition features. After training, the AE is then coupled to a Softmax activation function to detect AFib; a 96.0% accuracy was achieved.

### 2.3. For Real-Time Implementation

Detecting AFib in real-time is challenging, requiring algorithms that are highly sensitive and specific while also being robust to noise and artifacts, with low computational requirements. Additionally, designing embedded systems for AFib detection must take into account hardware constraints, such as memory allocation and processing power, as well as the efficiency and number of operations of the algorithm.

To address these challenges, Chen and colleagues [30] proposed an edge computing framework for AFib detection based on an embedded platform that mainly uses feature engineering to collect ECG signals in real-time. The model used for AFib detection is lightweight and achieved an F1-score of about 90% with only 18 multiplications and 18 additions. Directly analyzing data at the edge can reduce the response time, making the proposed solution feasible for analysis and training on the edge.

In addition, Andersen et al. [31] proposed a DL-based approach for real-time automatic detection of AFib in long-term ECG recordings. Their proposed model, a combination of CNNs and RNNs, achieved high sensitivity and specificity of 98.98% and 96.95%, respectively, after being trained and validated on three different databases with a total of 89 subjects. The model also demonstrated computational efficiency, analyzing 24 h of ECG recordings in less than one second. The proposed algorithm was tested on unseen datasets, resulting in 98.96% and 86.04% for specificity and sensitivity, respectively, and was found to outperform existing state-of-the-art models evaluated on standard benchmark ECG datasets. By learning data-driven features to distinguish AFib from the remaining rhythms in the signals, the proposed method eliminates the need for traditional feature engineering.

## 3. Methods

We study the ability to promptly distinguish ECG beats in NSR and in AFib using waveform morphology, for which an AE-based approach is proposed. Here, we briefly describe the purpose of the AE training and how classification is performed using the compressed version of the ECG waveforms.

### 3.1. Autoencoder-Based Models

**Figure 3:**
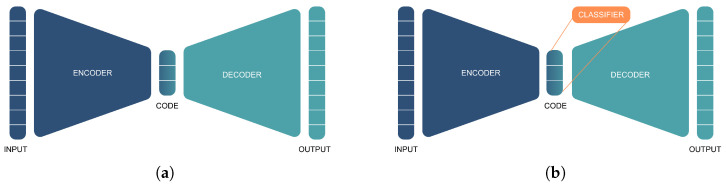
(**a**) A traditional autoencoder which is trained to reproduce the input samples, and (**b**) a supervised autoencoder that receives additional feedback from labeled samples.

An AE is an ANN that is divided into two elements: an encoder and a decoder (Figure 3a). The encoder is responsible for generating a feature vector (also called code) from the input, generally by compression, while the decoder is responsible for reconstructing the input from the feature vector. The traditional AE model (called undercomplete) achieves compression and reconstruction by, respectively, reducing and increasing the number of nodes layer by layer, often symmetrically. By enforcing the input to be compressed into a latent representation, and by using a cost function that favors the output to be as close as possible to the input (e.g., mean squared error (MSE)), the feature vector within the code should retain the most relevant information about the data’s nature, provided that the model converged and properly fit the data.

SAEs, on the other hand, take advantage of setting some nodes to zero, which can have a positive effect when learning an internal representation. Instead of reducing the number of nodes to achieve an information bottleneck, SAEs try to enhance the generalization ability by applying an L1 penalty to the code layer, which equals the sum of the absolute values of the ANN’s weights [32].

Another approach is to add a classifier in the AE’s bottleneck (Figure 3b), called a Supervised Autoencoder [33]. Although a regular AE is supervised in the sense that the target is the input, the set of features generated by the SupAE is regulated by both the MSE and the classification loss, which should result in higher classification performances.

**Figure 4:**
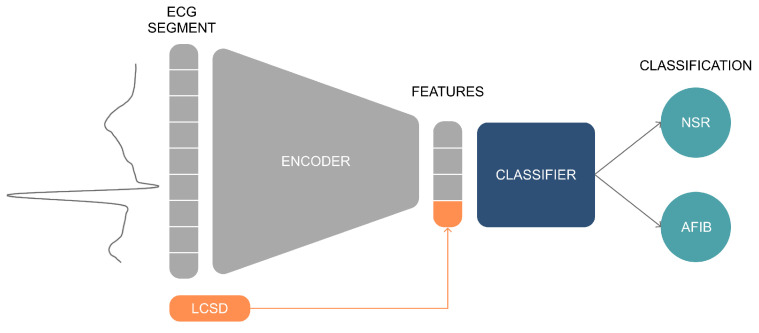
The proposed model for classifying ECG heartbeat waveforms into Normal Sinus Rhythm (NSR) and Atrial Fibrillation (AFib). It consists of an encoder responsible for extracting morphological features from the ECG segments, which are then used by a classifier. The LCSD metric is also used to train the classifier to provide local rhythm information and improve classification performance.

Upon a previously made systematic study of different combinations of AE type and classifier [34], the proposed model for AFib detection consists of first training a three-layer SAE (with 75% compression level) using ECG waveforms from both classes (NSR and AFib), and then using the encoded features as an input to a Multilayer Perceptron (MLP) binary classifier (Figure 4). By doing this, the ECG waveforms are mapped into a compressed version, consisting of feature vectors, which are then classified as NSR or as AFib.

### 3.2. Rhythm Feature

**Figure 5:**
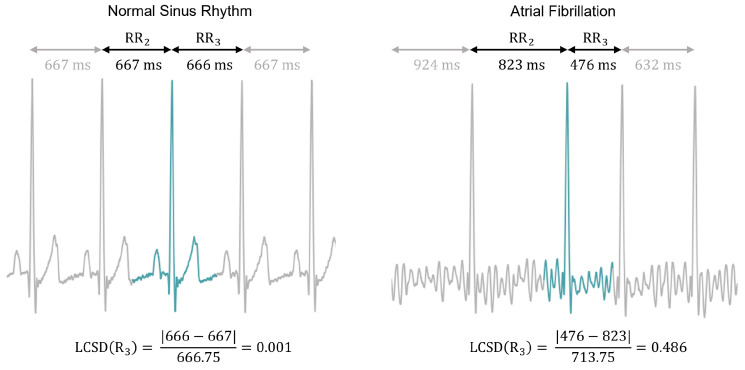
Example of LCSD values for ECG beats in NSR (left) and in AFib (right). Since RR-Intervals are more regular in NSR, their consecutive differences are smaller compared to AFib.

Since rhythm information (i.e., RR-Interval variability) is not fed into the AE, an additional feature was also tested to possibly enhance the classification task. This engineered feature is called Local Change of Successive Differences (LCSD) [34], which was specifically designed for the beat-by-beat classification problem. Given an ECG signal with R-peaks at time instants $R_{1},R_{2},...,R_{i},...,R_{N}$, the LCSD is computed for a given $R_{i}$ as:

(1) $$\mathrm{LCSD}\left(R_{i}\right)=\frac{\left|\left(R_{i+1}-R_{i}\right)-\left(R_{i}-R_{i-1}\right)\right|}{\frac{1}{N-1}\sum_{j=1}^{N-1}\left(R_{j+1}-R_{j}\right)},1<i<N,$$

which consists in computing the absolute difference between consecutive RR-Intervals and dividing by the mean RR-Interval of the recording. Because AFib is characterized by irregular RR-Intervals, the LCSD values in this rhythm should be different from the ones in NSR as illustrated in Figure 5.

## 4. Results

In this section, we first describe the datasets chosen to evaluate the performance of the proposed approach, then validate the preprocessing steps, including the LCSD feature calculation, and finally present the training and classification results.

### 4.1. Databases

In the last decades, a number of databases for AFib detection have been made publicly available to promote the development and testing of new algorithms. The AFDB [21,22], created by Boston’s Beth Israel Hospital laboratories and the Massachusetts Institute of Technology, contains 10-hour recordings of 25 people with AFib (mainly paroxysmal). The dataset contains annotations for NSR, AFib, atrial flutter, and AV junctional rhythm. The 250 Hz Holter recording system consisted of two ECG leads, however, with no indication of the electrodes’ placement.

Another well-known and recent database originated from the CinC2017 [23], which is considered to be one of the main sources of new publications related to AFib detection algorithms in 2018 (Figure 2) [19]. In addition, because of its acquisition environment, the CinC2017 database is particularly interesting to test algorithms using short-term ECG acquisitions from wearable devices. There are 12,186 single-lead recordings in this database, ranging in length from 9 to 60 s. The recordings are labeled as NSR, AFib, other rhythms, and noisy acquisitions. The data were collected using AliveCor’s single channel ECG devices, including the AliveCor^®^ KardiaMobile, with a Left Arm–Right Arm lead configuration (equivalent to Lead I [16]). The acquisitions were made using a sampling rate of 300 Hz with a 0.5–40 Hz bandwidth. More information regarding the aforementioned databases can be found in Table 1.

### 4.2. Preprocessing

**Figure 6:**
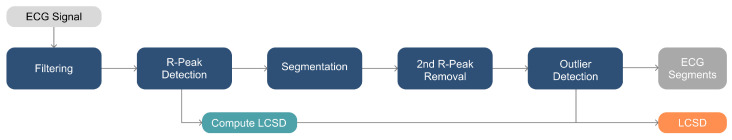
Preprocessing steps of the ECG recordings.

Data preprocessing (Figure 6) was conducted using the BioSPPy toolbox [35] on a Python 3 environment. All ECG signals were first filtered using a high-pass Finite Impulse Response (FIR) filter with a cut-off frequency of 0.5 Hz. An R-peak detection algorithm that follows the approach proposed by Hamilton [36] was applied to the CinC2017 data, and the AFDB R-peak data (already provided) were corrected to achieve more accurate R-peak locations. Only the first channel of the AFDB database (“ECG1”) was used.

**Figure 7:**
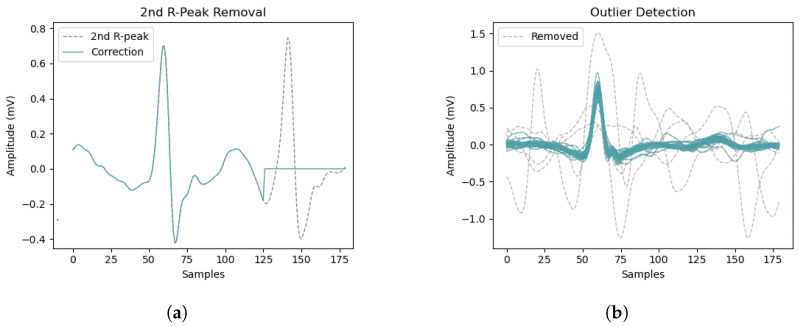
(**a**) Example of second R-peak removal from an AFib ECG segment using zero-padding. (**b**) Use of DMEAN to detect outliers in a set of ECG segments from a recording—valid segments are depicted as solid lines, while outliers are displayed in dashed lines. Data from the CinC2017 database [23].

Afterwards, all ECG recordings were segmented by beats using a segmentation technique proposed by Lourenço et al. [37], where ECG beats are obtained by clipping around the R-peaks (200 milliseconds (ms) before and 400 ms after). However, since this method generates fixed-length templates, second R-peaks can appear due to the short RR-Intervals caused by AFib. To prevent the model from being affected by such second R-peaks, they were removed by applying zero-padding 70 ms before their appearance, to also cover the Q-wave (Figure 7a).

In addition, to prevent the models from being trained with anomalous waveforms, an ECG outlier detection algorithm based on DMEAN was used [38], which discards ECG beats by their cosine distance to the average waveform (Figure 7b).

The number of NSR and AFib signal portions and beats extracted from the recordings is presented in Table 2.

### 4.3. Validation of the LCSD Metric

**Figure 8:**
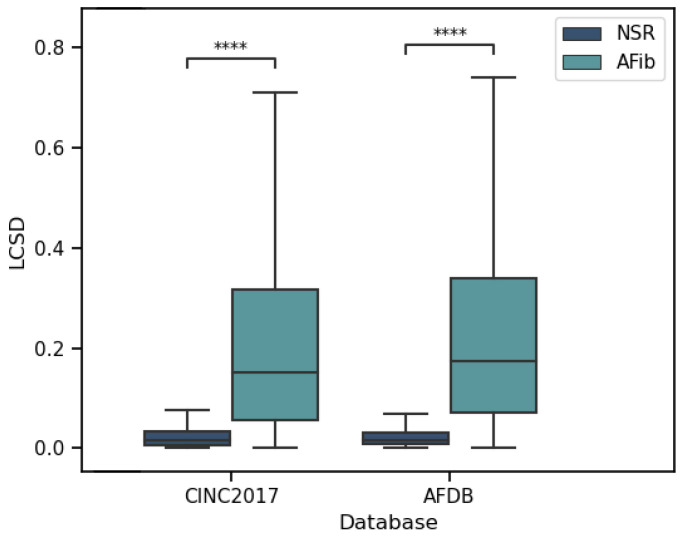
Distribution of LCSD values for NSR and AFib in two databases. The Mann–Whitney U test revealed significant statistical differences between the distributions, with p-values smaller than 0.0001 (marked by ****).

As previously presented, the LCSD feature was proposed to provide the model with local rhythm information. This metric is proportional to the absolute difference between consecutive RR-Intervals around an R-peak, which is expected to result in different values for NSR and AFib. To validate its usefulness, the Mann–Whitney U test was conducted on all databases, and the results showed significant differences between the two groups (as illustrated in Figure 8).

Additionally, the use of the LCSD metric was studied as the sole feature for the separation of NSR and AFib. A logistic regression classifier was trained to predict the classes and its performance was evaluated using various metrics, as reported in Table 3. The results show good performance, which highlights the importance of the LCSD metric to enhance the classification results of the morphology-based models.

### 4.4. Training

For both datasets, the data split consisted of using 80% of the signal portions for training and 20% for testing, and, to determine the number of epochs to train the AE models, a 10-fold cross-validation procedure was used. Because the AFDB is organized by patients, an additional data split approach was tested: 80% of the patients (18) for training and 20% for testing (5). This stratification by patient could give insight into whether the proposed model is able to generalize for ECG recordings of unseen patients.

After extraction of the ECG waveforms from each subject’s recording(s), the number of NSR samples used to train the models was limited by the number of AFib waveforms (i.e., undersampling of the majority class), to deal with class imbalance (Table 2).

The training procedure was performed using a TensorFlow 2 version in a Python 3 environment. The training data were standardized using the StandardScaler implementation of the Scikit-Learn Python library [39], and the validation and test inputs were transformed using the training standardization.

The loss functions chosen were MSE loss for the AEs and binary cross-entropy for the MLP classifiers. A maximum of 2000 epochs was set for all models, using an early stopping condition with a 50 epoch-patience after no decrease in validation loss. The models were trained on batches of 32 samples using the Adam optimizer algorithm with an initial learning rate set to $1\times 10^{-4}$. The architecture of the models can be found in Table A1 and detailed information regarding the training procedure is in Table A2.

**Figure 9:**
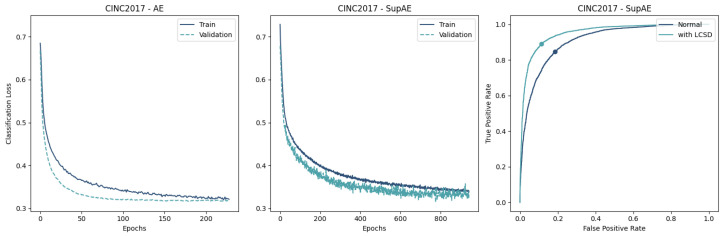
Comparison of Encoder-MLP (**left**) and Supervised Autoencoder (**center**) on CinC2017 data shows that SupAEs have insignificant changes in convergence and performance in terms of lower classification losses. The ROC curve of SupAE with and without the LCSD metric (**right**) highlights the best threshold value.

**Figure 10:**
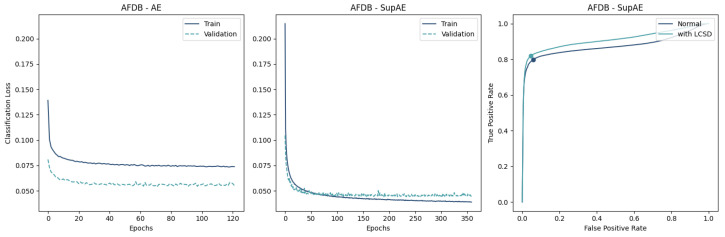
Comparison of Encoder-MLP (**left**) and Supervised Autoencoder (**center**) on AFDB data shows the SupAE’s quicker convergence and better performance in terms of lower classification losses. The ROC curve of SupAE with and without the LCSD metric (**right**) highlights the best threshold value.

**Figure 11:**
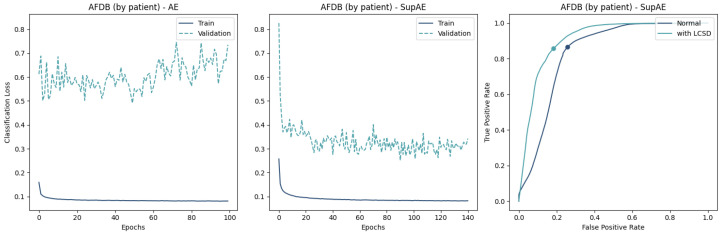
Comparing the performance of Encoder-MLP and Supervised Autoencoder on AFDB data using a patient-based split. The learning curves on the (**left**) and (**center**) show that both models face difficulties in converging the validation loss, possibly due to the presence of unseen pathological waveforms. The ROC curve of the SupAE model (**right**) with and without the LCSD metric highlights the best threshold value.

The resulting learning curves are represented in Figure 9, Figure 10 and Figure 11, where the differences in the convergence between the AE and SupAE models can be noted. Table A3 reports the training results of the models, with indication of the number of epochs, losses, and classification thresholds.

### 4.5. Classification

Table 4 summarizes the classification results using data from the CinC2017 and AFDB databases, including the models using patient-stratified data. The accuracy, F1-score, and Area Under Curve (AUC) are presented for the AE and SupAE models with and without the LCSD feature. The remaining metrics such as precision and recall are available in Table A4.

Because the training approach for the AEs and MLPs involved a 10-fold cross-validation, the instance of the models chosen for testing was the one with the lowest validation loss.

## 5. Discussion

The results of our study suggest that coupling a trained encoder with a classifier can effectively distinguish NSR from AFib waveforms using single-lead ECG recordings. The Supervised AEs demonstrated the ability to create a feature space that enhanced the classification performance, outperforming the “unsupervised” versions (Table 4). This is also supported by the lower train and validation losses (Figure 9, Figure 10 and Figure 11).

Since the features created by AEs are based only on ECG waveforms and were able to obtain high scores (86% F1-score with the SupAE), this suggests that morphological features alone are a distinct and sufficient factor for detecting AFib in ECG recordings. This is an advantage comparatively to most state-of-the-art algorithms that only use rhythm information (i.e., ventricular features), because these rely on the analysis of heartbeat variations occurring over a longer time period than a single ECG waveform.

In this respect, multiple-beat classification could potentially lead to superior performance. However, for the time being, existing datasets do not allow more detailed analysis, since the available labels are assigned to a signal portion rather than to individual beats. As a result, a signal portion labeled AFib may actually contain normal ECG beats (i.e., with a regular P-wave). This could have prevented the AE from reaching its full potential, as there could be mislabeled data.

Nevertheless, the SupAE with the LCSD metric achieved peak F1-scores of 88.8% and 87.9% for the CinC2017 and AFDB data, respectively, corresponding to a 6% and 2% increase in classification performance (Table 4). These improvements are supported by the validation tests of the LCSD metric (Section 4.3).

**Figure 12:**
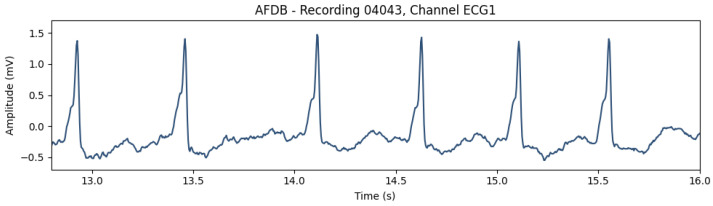
Portion of a pathological ECG waveform from the AFDB database (recording 04043).

Regarding the learning of the AE-based models, the difference between the two data-split approaches provides some insight into the generalization ability of the proposed approach. While the validation loss curve converged similarly to the training loss in the CinC2017 and AFDB training (Figure 9 and Figure 10), the AFDB stratified by patients did not, with high cross-entropy values (Figure 11). Although the models using this approach obtained F1-scores between 74% and 84% with the test data (Table 4), the training curves suggest that the model is not able to fully capture the ECG morphological differences of different patients. Indeed, some recordings of this database appear to have pathological ECG waveforms (Figure 12).

This observation clarifies the ability of AEs to be used in AFib detection. Since ECG morphology is unique for each subject, the use of AEs for patient-independent diagnosis is limited. In this respect, algorithms using RR-Intervals to perform AFib detection have an advantage over morphology-based ones, as they have a greater generalization ability. On the other hand, the AE-based approach for subject-specific AFib detection yields good results.

Table 5 compares the performance of our approach to other state-of-the-art methods for AFib detection. One of the key aspects to highlight is that our method achieved competitive F1-scores when compared to the other approaches that were designed for real-time implementation, despite not using any feature engineering. Our approach extracts useful features automatically from the input data, making it more efficient and scalable.

For the CinC2017 dataset, our approach achieved an F1-score of 88.8% (with 180 trainable parameters), which is slightly lower than the F1-score obtained by Chen et al. [30] using an MLP (with 8 trainable parameters) with feature engineering (91.5%). However, it is worth noting that feature extraction may involve preprocessing steps such as interpolation, frequency analysis, and other tasks that may compromise latency and be more susceptible to noise. Zhang et al. [27] achieved a higher F1-score of 99.0% using DenseNet+Bi-LSTM, but their approach, which involves a significant number of operations (e.g., convolutions, multiplications, additions), may not be suitable for real-time and resource-limited systems.

Regarding the AFDB dataset, our approach achieved an F1-score of 87.9%, which is comparable to the F1-score achieved by Zhang et al. [27] using DenseNet+Bi-LSTM (87.6%) but lower than the F1-score obtained by Andersen et al. [31] using CNN+LSTM (97.2%). Nevertheless, the approach by Andersen et al. uses a more complex architecture (with 159,841 trainable parameters), which may not be optimal for AFib real-time detection. In contrast, our approach is simple and efficient, making it compatible with real-time implementation.

Compared to DL methods, our proposed approach offers several advantages. Firstly, our model has a significantly simpler architecture, with only 148 trainable parameters for AFDB and 180 for CinC2017, as opposed to the thousands or even millions of parameters required by DL models. This makes our approach faster and more computationally efficient, which is important for real-time detection applications. Secondly, our model is more interpretable, as the extracted features are directly related to the waveform morphology. In contrast, DL models often rely on complex and opaque architectures, making it difficult to explain how decisions are made. Lastly, our approach does not require extensive feature engineering, which is often a time-consuming and challenging task in ML/DL applications.

Based on the overall score of the proposed morphological AE-based model, our work demonstrates the ability of AEs to automatically extract features tailored to a specific classification task, with performance that compares favorably to other state-of-the-art algorithms. Furthermore, because of their efficient feature mapping, AEs avoid the need for explicit feature extraction, and can be easily implemented in resource-limited and real-time systems, which is especially relevant in wearable devices that aim to provide real-time results and/or that do not rely on external processing units (e.g., cloud computing). The low number of trainable parameters and operations, as well as the extracted morphological features, offer advantages over DL models. However, some limitations have been identified regarding the generalization ability of morphology-based algorithms.

## 6. Conclusions

In this work we evaluate an AE-based technique to classify ECG beats into NSR and AFib. The results of combining a trained encoder with a classifier reveal that single-lead ECG recordings can be used to differentiate NSR and AFib waveforms. In general, SupAEs can provide a feature space that improves classification performance as they produce better results.

This approach was tested with two highly referenced databases that demonstrate the ability of AEs to handle different acquisition modalities. Namely, the AFDB database, containing ECG recordings from hospital-grade Holter devices, and the CinC2017 database with single-lead ECG data recorded with a mobile device using current technology. Since the latter results from the subject’s interaction with a device, its use case is more naturalistic.

This versatile strategy of using single-lead ECG beats for near real-time classification is an advantage over state-of-the-art algorithms that only use rhythm information and/or need a longer time period to be computed. However, using the short-term feature LCSD greatly enhances classification performances. The best model achieved F1-scores of 88.8% and 87.9% using the data from the CinC2017 and AFDB. However, when a patient-based data split was used (i.e., the model was evaluated using data from patients that were left out during training), the F1-score dropped to 84%. This suggests that morphology-based AFib detection models based on Supervised Autoencoders generalize better when trained with patient-specific data.

The performance of our approach for AFib detection was compared to other state-of-the-art methods, and it was found to achieve competitive F1-scores without requiring any feature engineering. The approach extracts useful features automatically from input data, making it more efficient and scalable. It is also simpler and more efficient compared to DL methods and suitable for real-time detection. To the best of our knowledge, this is the first work that presents a near real-time morphological approach to detect AFib under naturalistic ECG acquisition with a mobile-based device.

Future work may explore the extension of the proposed technique to classify multiple arrhythmias, beyond NSR and AFib, to enhance the model’s clinical relevance. In addition, investigating the use of AEs to capture the morphological features of other types of cardiac pathologies could further expand the proposed approach’s applicability. A next step for future research could also involve the integration of the proposed technique into a real-time ECG monitoring system. This would require addressing the challenge of deploying a computationally efficient model that does not compromise classification performance while accounting for device and data transmission constraints.

## Figures and Tables

**Table 1:** Description of publicly available databases for atrial fibrillation detection.

Database (Year)	Lead System	Duration Recording	Sampling Rate (Hz)	ADC Resolution	Dynamic Range	Bandwidth
CinC2017 (2017)	Single-lead	9–60 s	300	16-bit	±5 mV	0.5–40 Hz
AFDB (1983)	Two-lead	10 h	250	12-bit	±10 mV	0.1–40 Hz

**Table 2:** Number of recordings, signal portions, and ECG waves after data preprocessing.

Database	Recordings	Signal Portions		ECG Waves	
		NSR	AFib	NSR	AFib
CinC2017	5788	5050	738	144,310	27,969
AFDB	23	288	289	478,898	365,455

**Table 3:** Performance of the Logistic Regression classifier for the LCSD metric in two databases.

Database	Accuracy	Precision	Recall	F1-Score
CinC2017	0.870	0.739	0.304	0.431
AFDB	0.757	0.820	0.562	0.667

**Table 4:** Classification metrics for the proposed AE-based models using data from two PhysioNet databases. The best models for each database are highlighted in bold (based on the F1-Score).

Database	SupAE	Features	Accuracy	F1-Score	AUC
CinC2017	No	AE	0.820	0.827	0.892
		AE+LCSD	0.885	0.886	0.945
	Yes	AE	0.831	0.834	0.908
		AE+LCSD	0.888	0.888	0.951
AFDB	No	AE	0.777	0.803	0.797
		AE+LCSD	0.818	0.837	0.825
	Yes	AE	0.870	0.860	0.874
		AE+LCSD	0.887	0.879	0.908
AFDB (by patient)	No	AE	0.703	0.746	0.719
		AE+LCSD	0.732	0.763	0.754
	Yes	AE	0.804	0.815	0.833
		AE+LCSD	0.837	0.840	0.907

**Table 5:** Classification performances of different ANN-based algorithms, and comparison with our results.

Data Source	Author	Methodology	F1-Score	For Real-Time	Feature Engineering
CinC2017	Zhang et al. [27]	DenseNet+Bi-LSTM	0.990	No	No
	Chen et al. [30]	MLP	0.915	Yes	Yes, 22 features
	Proposed Approach	AE+MLP	0.888	Yes	No
AFDB	Zhang et al. [27]	DenseNet+Bi-LSTM	0.876	No	No
	Andersen et al. [31]	CNN+LSTM	0.972	Yes	No
	Proposed Approach	AE+MLP	0.879	Yes	No

## Data Availability

The data presented in this study are openly available in Physionet at https://physionet.org/content/challenge-2017 (accessed on 30 January 2021 ) and https://doi.org/10.13026/C2MW2D (accessed on 30 January 2021).

## Appendix A

**Table A1:** Architecture of the AE and MLP models for CinC2017 and AFDB Databases. The SupAE consists of training the AE+MLP model. In models with the use of LCSD, the MLP is increased in one input node.

Database	Model	Layer	Size	Activation Function	Regularization
CinC2017	AE	Input	180	-	-
		Dense	45	Linear	L1 ($1\times 10^{-5}$)
		Dense	180	Linear	L1 ($1\times 10^{-5}$)
	MLP	Input	45	-	-
		Dense	45	ReLU	0.2% Dropout
		Dense	45	ReLU	0.2% Dropout
		Dense	45	ReLU	0.2% Dropout
		Output	1	Sigmoid	-
AFDB	AE	Input	150	-	-
		Dense	37	Linear	L1 ($1\times 10^{-5}$)
		Dense	150	Linear	L1 ($1\times 10^{-5}$)
	MLP	Input	37	-	-
		Dense	37	ReLU	0.2% Dropout
		Dense	37	ReLU	0.2% Dropout
		Dense	37	ReLU	0.2% Dropout
		Output	1	Sigmoid	-

**Table A2:** Training details for the Autoencoder and MLP Classifier models.

	Parameter	Autoencoder	MLP Classifier
Training	Loss Function	Mean Squared Error	Binary Cross-Entropy
	Epochs	2000	2000
	Early Stopping	min mode, 50 epoch-patience	min mode, 50 epoch-patience
	Batch Size	32	32
Optimizer	Type	Adam	Adam
	Learning Rate	$1\times 10^{-4}$	$1\times 10^{-4}$
	Beta1	0.9	0.9
	Beta2	0.999	0.999
	Epsilon	$1\times 10^{-7}$	$1\times 10^{-7}$

## Appendix B

**Table A3:** Training results for the AE-based models using data from two PhysioNet databases.

Database	SupA E	Features	Num. of Epochs	Training Loss (min)	Validation Loss (min)	AUC Threshold
CinC2017	No	M	229	0.320	0.317	0.337
		M+LCSD	223	0.218	0.217	0.408
	Yes	M	942	0.337	0.314	0.46
		M+LCSD	2000	0.195	0.214	0.439
AFDB	No	M	122	0.074	0.054	0.298
		M+LCSD	206	0.067	0.05	0.563
	Yes	M	359	0.039	0.044	0.239
		M+LCSD	2000	0.035	0.039	0.17
AFDB (by patient)	No	M	100	0.080	0.491	0.313
		M+LCSD	55	0.082	0.373	0.437
	Yes	M	141	0.081	0.251	0.450
		M+LCSD	2000	0.076	0.107	0.480

**Table A4:** Classification metrics for the proposed AE-based models using data from two PhysioNet databases.

Database	SupAE	Features	Accuracy	Precision	Recall	F1-Score	AUC
CinC2017	No	M	0.82	0.794	0.863	0.827	0.892
		M+LCSD	0.885	0.876	0.897	0.886	0.945
	Yes	M	0.831	0.821	0.847	0.834	0.908
		M+LCSD	0.888	0.888	0.889	0.888	0.951
AFDB	No	M	0.777	0.718	0.911	0.803	0.797
		M+LCSD	0.818	0.759	0.932	0.837	0.825
	Yes	M	0.87	0.933	0.798	0.86	0.874
		M+LCSD	0.887	0.947	0.82	0.879	0.908
AFDB (by patient)	No	M	0.703	0.652	0.871	0.746	0.719
		M+LCSD	0.732	0.684	0.864	0.763	0.754
	Yes	M	0.804	0.771	0.865	0.815	0.833
		M+LCSD	0.837	0.824	0.857	0.84	0.907
